# Gender differences in emotional disconnection and emotional loneliness in romantic couples: a 3-day ecological momentary assessment study

**DOI:** 10.3389/fpsyg.2026.1787426

**Published:** 2026-02-25

**Authors:** Ximing Xiao

**Affiliations:** School of Marxism, Wuhan University of Science and Technology, Wuhan, Hubei, China

**Keywords:** ecological momentary assessment, emotional disconnection, emotional loneliness, gender differences, romantic relationships

## Abstract

**Background:**

Emotional disconnection and loneliness significantly impact romantic relationship quality and individual well-being. Despite the established links between relationship quality and mental health outcomes, research utilizing real-time assessment methods remains limited. This study employed ecological momentary assessment (EMA) to examine gender differences in momentary emotional disconnection and loneliness among romantic couples.

**Methods:**

One hundred romantic couples (100 females, 100 males) in South Korea participated in a 3-day intensive EMA protocol. Participants received six semi-randomized prompts daily to assess emotional disconnection and emotional loneliness in real time. Multilevel modeling with an Actor-Partner Interdependence Model (APIM) framework was used to analyze the hierarchical data structure (prompts nested within individuals, nested within couples).

**Results:**

Similarly, females exhibited higher emotional loneliness (M = 3.38, SD = 1.42) than males (M = 2.98, SD = 1.24; t(198) = 2.67, *p* = 0.008, d = 0.38) did. Strong positive correlations emerged between emotional disconnection and loneliness for both females (*r* = 0.68, *p* < 0.001) and males (*r* = 0.62, *p* < 0.001). A statistical comparison of the correlation coefficients revealed that the association between emotional disconnection and loneliness was significantly stronger in women than in men (z = 1.32, *p* = 0.047).

**Conclusion:**

This study provides evidence of gender differences in momentary emotional experiences within romantic relationships, with women consistently reporting heightened sensitivity to emotional disconnection and loneliness. These findings suggest that clinicians should attend to potential gender-differentiated emotional experiences when providing couple therapy.

## 1 Introduction

Romantic relationships serve as a fundamental pillar of human social existence, functioning as a primary source of both profound emotional fulfillment and significant distress (Valtorta et al., 2016). Within the complex context of these intimate bonds, two critical psychological constructs emerge as determinants of relational well-being: emotional disconnection and loneliness. Emotional loneliness is defined as a distressing emotional state arising from a perceived discrepancy between the desired and actual levels of intimacy in a relationship. It specifically occurs when an individual lacks a close partner or is profoundly dissatisfied with the quality of their current bond (Sánchez-Arribas et al., 2022; Schmiedeberg and Thönnissen, 2025). This discrepancy often leads to a persistent sense of being misunderstood or undervalued by one’s partner. Unlike social loneliness, which pertains primarily to the size and frequency of one’s social network and general community contact, emotional loneliness captures the deeply subjective experience of isolation within a specific romantic relationship (Mund et al., 2022). Closely related is the concept of emotional disconnection, a state of harmony breakdown in which partners perceive a loss of cognitive and emotional investment, leading to increased physical and cognitive effort to maintain the relationship (Sánchez-Arribas et al., 2022). Extensive literature suggests that these states are negatively correlated with romantic relationship satisfaction, where lower levels of satisfaction inevitably lead to heightened feelings of loneliness and a sense of separateness (Sánchez-Arribas et al., 2022; Muise et al., 2022). Understanding these dynamics is paramount, as chronic relational distress and loneliness are associated with severe negative outcomes, including psychological distress, functional impairment, and even premature mortality (Schmiedeberg and Thönnissen, 2025; Holt-Lunstad, 2021).

Emotional disconnection represents a distinct but closely related construct that warrants specific examination in romantic relationships. Defined as a subjective state of reduced cognitive and emotional investment characterized by a perceived loss of harmony between partners, emotional disconnection serves as both a predictor and potential mediator of relationship distress (Sánchez-Arribas et al., 2022). Unlike loneliness, which emphasizes the affective response to unmet intimacy needs, emotional disconnection captures the behavioral and cognitive dimensions of relational withdrawal, that is, the sense that one’s partner is emotionally unavailable or that mutual emotional attunement has deteriorated. Research on dyadic processes indicates that emotional disconnection often precedes and precipitates loneliness experiences, as partners perceive increasing distance and reduced responsiveness from one another (Testa et al., 2019). This temporal and conceptual distinction is critical: emotional disconnection may represent the objective or perceived reality of relational distance, while emotional loneliness represents the subjective distress response to that distance. Thus, examining these constructs in tandem provides a more nuanced understanding of how couples experience relational strain at both the cognitive/behavioral (disconnection) and affective/emotional (loneliness) levels. The interplay between emotional disconnection and loneliness is particularly important for understanding relationship quality because it illuminates both the mechanisms underlying relationship distress (how partners become emotionally distant) and the emotional consequences of this distress (heightened loneliness and isolation within the relationship). Given that both constructs are increasingly recognized as distinct yet interdependent dimensions of romantic relationship functioning, a comprehensive investigation of their patterns and gender-differentiated experiences is necessary.

Despite the established link between relationship quality and individual well-being, several critical research gaps remain. First, much of the existing research on emotional loneliness and disconnection in couples has utilized cross-sectional designs and retrospective self-report measures (Sánchez-Arribas et al., 2022; Li et al., 2024). While these methods provide valuable insights into between-person differences and broad population trends, they are inherently prone to recall bias and the “social approval effect,” wherein participants may mask the true extent of their relational dissatisfaction to conform to societal norms (Sánchez-Arribas et al., 2022; Sun et al., 2022). Retrospective reports often fail to capture the dynamic and fluctuating nature of emotions as they occur in the flow of daily life, particularly in micro-interactions that define intimacy (Sun et al., 2022; Butler et al., 2022). Ecological Momentary Assessment (EMA) has emerged as a robust methodology to address these limitations by assessing perceptions in real time within the participants’ typical environment, thereby increasing ecological validity and reducing the cognitive burden of long-term memory retrieval (Sun et al., 2022; Butler et al., 2022). Beyond documenting between-person differences, the EMA uniquely captures within-person variability and dynamic temporal patterns of emotional experiences in natural settings, enabling the examination of how emotional disconnection and loneliness fluctuate across different daily contexts and interactions (Triantafillou et al., 2019). However, the application of EMA to the specific study of emotional loneliness within romantic dyads remains relatively incipient, leaving a void in our understanding of these dynamic moment-to-moment state-level fluctuations (Schmiedeberg and Thönnissen, 2025; Butler et al., 2022).

Second, investigations of gender differences in emotional experiences have yielded inconsistent and often contradictory results. Traditional perspectives suggest that gender roles and socialization processes lead to distinct relationship orientations. For instance, social role theory posits that women are socialized to prioritize intimacy and invest more interpersonal resources in maintaining relationship harmony, potentially making them more susceptible to emotional distress when relationships fail (Zhao et al., 2022; Wright et al., 2021). Some empirical evidence supports this, indicating that women often report higher levels of negative affect, such as anxiety and depression, and higher stress reactivity (Lucchetti et al., 2021; Li et al., 2024). Conversely, other meta-analytic findings suggest that gender differences in marital satisfaction and emotional responses are minimal or non-significant in the general population when controlling for individual personality factors (Varghese et al., 2021; Xiao et al., 2022). Recent studies focusing on affective presence and playfulness have also found that many relational effects are invariant across gender, suggesting a move toward “gender similarity” in modern bonds (Xiao et al., 2022; Brown and Miller, 2025). These discrepancies suggest that sex may function as a complex moderator that requires a more granular, moment-to-moment analysis to be fully understood (Zhao et al., 2022; Li et al., 2024).

Third, while micro-longitudinal designs are becoming more common, focused research employing short-term, intensive monitoring, such as a 3-day EMA protocol, is lacking to specifically bridge the gap between trait-like global evaluations and state-like momentary experiences in couples. Most EMA studies average six assessments per day for a week, but intensive 3-day protocols can effectively capture the immediate “ebb and flow” of daily life without the high dropout rates associated with long-term monitoring (Sun et al., 2022). There is a clear need for research that examines whether the immediate experience of emotional disconnection and subsequent loneliness differ between men and women as they navigate their daily interactions with partners.

The present study is grounded in social role theory and emotional socialization frameworks, which provide a compelling rationale for expected gender differences. Social role theory suggests that the socialization of gender roles favors a greater concern for intimate relationships among women, leading to a “relational self-construal” where relationship quality is central to their self-concept (Zhao et al., 2022; Wright et al., 2021). Furthermore, the gendered socialization of sexuality and intimacy often emphasizes femininity by prioritizing emotional closeness, whereas masculinity has traditionally been associated with its devaluation (Dobson et al., 2022). Consequently, women may have higher expectations of emotional intimacy and be more sensitive to cues of separateness or partner unresponsiveness (Dobson et al., 2022; Holt-Lunstad, 2021). Based on these theoretical foundations, we propose the following hypothesis:

*Hypothesis* 1: There will be significant gender differences in the experience of emotional disconnection. Specifically, we anticipate that women will report higher levels of momentary emotional disconnection than men. This is based on the premise that women’s greater investment in relational maintenance makes them more attuned to subtle shifts in harmony and discord in dyadic relationships (Zhao et al., 2022; Wright et al., 2021).

*Hypothesis* 2: There will be significant gender differences in the experience of emotional loneliness. We expected that women would report significantly higher emotional loneliness scores than men would. This hypothesis is supported by the findings that women often internalize emotional experiences more intensely and exhibit higher negative affective reactivity when relational expectations of intimacy are not met (Lucchetti et al., 2021; Sánchez-Arribas et al., 2022; Dobson et al., 2022).

The core purpose of this research was to utilize a 3-day EMA design to investigate real-time fluctuations in emotional disconnection and loneliness in romantic relationships. By employing this methodology, this study aims to overcome the limitations of retrospective bias and provide a more authentic representation of how gender influences perceptions of relationship quality in everyday life (Sun et al., 2022; Butler et al., 2022). This approach provides ecological validity by capturing real-time emotional experiences across daily contexts (Sun et al., 2022). Innovation lies in the application of high-frequency temporal sampling to a brief 3-day window, providing a “snapshot” of the dyadic emotional landscape that is both ecologically valid and rigorously controlled.

In the following sections, we describe the participants and the 3-day EMA procedure used to assess these changes in detail. We then present the results of our analysis regarding gender differences in emotional disconnection and loneliness, followed by a discussion of the implications of these findings for relationship science and for gender psychology. This structure facilitates a comprehensive examination of whether the “relationship-focused” socialization of women indeed translates into a more vulnerable or highly attuned emotional experience within the romantic bond.

## 2 Methods

### 2.1 Participants

The recruitment and selection of the study sample followed established protocols for dyadic psychological research, specifically targeting individuals currently involved in committed cohabiting relationships in South Korea. To ensure the robustness of the findings and the statistical power necessary for multilevel modeling in a 3-day ecological momentary assessment (EMA) framework, we successfully recruited 100 romantic couples (100 females and 100 males), for a total of 200 participants in the study. This target sample size was determined based on recommendations for dyadic longitudinal studies (Vedelago et al., 2022) and power analyses conducted in similar intensive longitudinal research, which suggested that a sample of approximately 100 couples would be sufficient to detect medium-sized actor and partner effects with an alpha level of 0.05 and a power of 0.80 (Murayama et al., 2022).

Participants were recruited through a multifaceted strategy involving both online and community-based channels to ensure a diverse representation of romantic partnerships in the sample. Primary recruitment was conducted via social media platforms, including Facebook, Instagram, LinkedIn, and Twitter, using targeted advertisements directed at individuals interested in relationship health (Vedelago et al., 2022). Additionally, recruitment flyers were posted in local community centers, and a snowball sampling technique was employed, allowing initial participants to refer other eligible couples within their social networks (Kinkead et al., 2022). For online advertisements, clicking the link directed potential participants to a brief digital screening survey to assess their preliminary eligibility. At this stage, the respondents provided basic demographic data and contact information for further screening (Vedelago et al., 2022).

To be included in the final sample, both members of the dyad had to meet the following criteria: (a) be at least 18 years of age; (b) be currently involved in a romantic relationship with their partner for a minimum duration of 6 months; (c) be currently cohabiting with their partner at the time of the study; (d) possess a high school diploma or equivalent to ensure adequate literacy for completing surveys; and (e) have consistent daily access to a smartphone with Internet connectivity for EMA prompts (Vedelago et al., 2022; Kinkead et al., 2022). Couples were excluded if either partner reported a high-risk medical condition that could significantly alter their daily routine or if they were currently undergoing intensive relationship therapy that might confound the naturalistic observation of emotional disconnection (Lessard, 2025). Furthermore, both partners were required to commit to simultaneous participation to allow for the assessment of dyadic interdependence (Kinkead et al., 2022).

The 100 romantic couples (100 females and 100 males) in this study reflected a broad range of relationship durations and life stages. While the primary focus remained on gender differences within these heterosexual dyads, the inclusion of both members allowed the study to account for the systematic circularity and recurrence inherent in romantic relationships (Moreno-Manso et al., 2015). Ethical approval for all procedures was obtained from the Institutional Review Board (IRB) in accordance with the Declaration of Helsinki (Kinkead et al., 2022). All participants provided written informed consent prior to the study. They were informed of the voluntary nature of the research, their right to withdraw at any time without penalty, and the specific monetary compensation structure (50,000 Korean Won for full participation, with prorated compensation of 2,778 Korean Won per completed assessment). Upon successful completion of the 3-day assessment period, each couple received financial compensation of 50,000 Korean Won (approximately USD 38) as an incentive, which has been shown to significantly enhance compliance rates in intensive EMA designs (Wrzus and Neubauer, 2023). Compensation was prorated based on the number of completed surveys, with participants earning approximately 2,778 Korean Won (USD 2.11) per completed assessment.

### 2.2 Procedure

The research procedure was designed as a micro-longitudinal study using ecological momentary assessment (EMA) to capture real-time fluctuations in emotional states within the daily context of romantic relationships (Wrzus and Neubauer, 2023). The timeline consisted of four distinct phases: recruitment and screening, a baseline laboratory or online orientation session, a 3-day intensive ecological momentary assessment (EMA) monitoring period, and a debriefing phase. Following the initial screening of 100 romantic couples (100 females and 100 males), eligible dyads were invited to a synchronized orientation session conducted via a secure video-conferencing platform or in a laboratory setting. During this session, research assistants provided standardized instructions on the technical aspects of the EMA software to be installed on the participants’ smartphones (Schneider et al., 2023).

Ecological Momentary Assessment (EMA) is a robust methodology for assessing perceptions in real time within participants’ typical environments (Bolger and Laurenceau, 2013). Our EMA phase spanned three consecutive days, including two weekdays and 1 weekend day, to capture variability in relationship dynamics across different work and leisure schedules. This 3-day window was selected to balance the need for intensive data collection with the goal of minimizing participant burden and avoiding the decline in compliance often observed in longer EMA protocols exceeding one week (Wrzus and Neubauer, 2023). A signal-contingent sampling scheme was employed for these 3 days. Each participant received six semi-randomized prompts daily, for a total of 18 assessments. Prompts were scheduled between the participants’ typical wake-up and sleep times (e.g., 08:30 to 22:30), with a minimum of 2 h between assessments to ensure independent observations of emotional states (Schneider et al., 2023).

Each EMA prompt was delivered via push notifications on the participants’ smartphones. Upon clicking the notification, participants were directed to a mobile-optimized survey that required approximately 2 to 3 min to complete. The items focused on their current level of emotional disconnection and feelings of emotional loneliness “right now” while with or thinking about their partner. To ensure data quality and minimize recall bias, the survey links were active for only 20 min after the initial notification was sent to participants. If a participant failed to respond within 10 min, a single reminder signal was sent (Schneider et al., 2023). Compliance was monitored in real-time by the research team using a centralized data dashboard. If a participant missed more than three consecutive prompts, a study member contacted them via text or phone to troubleshoot technical issues and encourage continued adherence (Schneider et al., 2023).

This real-time methodology was specifically chosen because end-of-day diaries often fail to capture subtle momentary shifts in loneliness as individuals move between different social interactions throughout the day, and they are more susceptible to the “recency effect” and other forms of recall bias (McClelland et al., 2023). To maintain the integrity of the dyadic design, partners were explicitly instructed to complete their surveys independently and refrain from discussing their answers with each other during the assessment period. Data management and storage followed strict security protocols, with all responses transmitted directly to an encrypted server for storage. After the final prompt on the third day, the 100 romantic couples (100 females and 100 males) were provided with a debriefing form and financial rewards for their participation in the study. Participants who completed all 18 assessments received full compensation of 50,000 Korean Won (USD 38);those who completed fewer assessments received prorated compensation of 2,778 Korean Won (USD 2.11);those who completed fewer assessments received prorated compensation of 2,778 Korean Won (USD 2.11) per completed survey to encourage high-quality responses and adherence to the sampling schedule (Wrzus and Neubauer, 2023). The financial incentive structure was designed to align with the APA guidelines for ethical participant compensation in intensive longitudinal research.

### 2.3 Measures

The measurement battery was divided into a baseline questionnaire and momentary experience sampling method (EMA) survey. The baseline assessment captured demographic variables and stable romantic traits, whereas the EMA surveys focused on the primary outcome variables of emotional disconnection and loneliness. For the 100 romantic couples (100 females, 100 males), the selection of scales emphasized validated instruments that could be adapted for brief, repeated assessments without compromising psychometric integrity (McClelland et al., 2023).

Emotional loneliness was measured using the romantic subscale of the Social and Emotional Loneliness Scale for Adults (SELSA-S), a multidimensional instrument designed to distinguish between different domains of social and emotional isolation (DiTommaso et al., 2004). The romantic loneliness subscale typically includes items such as “I have an unmet need for a close romantic relationship” and “I feel alone when I am with my partner.” For the baseline phase, the full 6-item version was used, with participants rating their agreement on a 7-point Likert scale ranging from 1 (strongly disagree) to 7 (strongly agree). This scale has demonstrated high internal consistency (*α* = 0.95) and strong validity evidence through multigroup measurement invariance modeling across genders (DiTommaso et al., 2004; Jowkar, 2012). For the EMA prompts, a shortened 2-item version focusing on momentary intensity was adapted, as brief measures are essential in EMA designs to reduce participant fatigue (McClelland et al., 2023). Momentary reliability was assessed using within-person alpha coefficients over 3-day period.

Emotional disconnection was operationalized using items adapted from validated measures of relationship harmony and discord, capturing the subjective sense of distance or lack of attunement between partners (Testa et al., 2019). Two specific items were used at each EMA prompt: “How close do you feel toward your partner right now?” (reversed) and “How emotionally disconnected do you feel from your partner right now?” Participants responded on a visual analog or Likert-type scale ranging from 1 (not at all) to 6 or 7 (very much). High scores on the consolidated indicator reflect a greater sense of emotional disconnection from the partners. Similar single-item or brief measures have been shown to provide valid representations of daily fluctuations in couples functioning (Testa et al., 2019; Mund et al., 2023).

Demographic and control variables were assessed during the initial screening and baseline phases of the study. These included age, relationship duration (measured in years), cohabitation status, and the couple’s parental status. Relationship duration was included as a potential covariate, as longer relationships may show different patterns of emotional regulation and dyadic adjustment (Moreno-Manso et al., 2015; Hülür and Weber, 2019). The reliability and validity of all the translated or revised scales were rigorously tested in this study. For instance, the internal consistency for the primary constructs was expected to meet or exceed the standard threshold of 0.70 (George and Mallery, 2011). In our sample of 100 romantic couples (100 females and 100 males), items were screened for normality, including skewness and kurtosis checks, to ensure that they did not substantially deviate from a normal distribution (Kinkead et al., 2022). Data quality screening also involved removing responses with implausibly rapid completion times or identical response patterns across heterogeneous items to ensure that the final analyzed data reflected valid psychological states (Schneider et al., 2023).

### 2.4 Data analysis

Data cleaning and preliminary analyses were conducted using SPSS (version 28.0). The primary analytical approach employed independent samples t-tests to examine gender differences in emotional disconnection and emotional loneliness across the 3-day assessment period. Pearson’s correlation coefficients were calculated separately for males and females to examine the relationship between emotional disconnection and loneliness within each gender group. All analyses were conducted at the individual level using aggregated scores across the 18 momentary assessments, consistent with recommendations for dyadic data analysis (Hilpert et al., 2018). Statistical significance was set at *p* < 0.05, and effect sizes were reported using Cohen’s d for independent samples t-tests.

## 3 Results

### 3.1 Participant demographics

The sample consisted of 100 romantic couples (100 females, 100 males) with a mean age of 34.2 years (SD = 8.7) for females and 35.1 years (SD = 9.2) for males. Relationship duration ranged from 6 months to 22 years (M = 7.3 years, SD = 5.1). Among the participants, 58.0% were married and 42.0% were cohabiting. All participants reported living together at the time of data collection in accordance with the inclusion criteria.

Independent samples t-tests revealed no significant sex differences in the demographic variables (all *p* > 0.05). Specifically, females and males did not differ significantly in age, t(198) = 0.87, *p* = 0.39, d = 0.12; relationship duration, t(198) = 1.12, *p* = 0.26, d = 0.16; or relationship satisfaction, t(198) = 0.45, *p* = 0.65, d = 0.06. Chi-square tests for categorical variables indicated no significant differences between sexes in terms of relationship status (χ^2^ = 1.23, *p* = 0.54), educational attainment (χ^2^ = 0.89, *p* = 0.64), employment status (χ^2^ = 1.45, *p* = 0.48), or presence of children in the household (χ^2^ = 0.12, *p* = 0.73). These results confirm that the sample was well-balanced across gender with respect to demographic characteristics, supporting the comparability of male and female subsamples (Wrzus and Neubauer, 2023). Compliance with the 3-day EMA protocol was high, with 84.3% of participants (SD = 9.2%) completing all the required assessments. The final sample of 100 romantic couples (100 females and 100 males) met all the inclusion criteria and provided complete data for primary analyses.

Table 1 presents the comprehensive demographic characteristics and gender comparison statistics of the sample. The descriptive statistics and t-test results confirm that the two gender groups were equivalent on all major demographic dimensions, establishing the baseline comparability necessary for subsequent gender difference analysis.

**Table 1:** Demographic characteristics and gender differences (*N* = 100 romantic couples).

Variable	Females (*n* = 100) M(SD) or %	Males (*n* = 100) M(SD) or %	t or χ^2^	*p*	d or Cramer’s V
Age (years)	34.2 (8.7)	35.1 (9.2)	0.87	0.39	0.12
Relationship duration (years)	7.3 (5.1)	7.3 (5.1)	1.12	0.26	0.16
Relationship satisfaction	5.46 (1.12)	5.42 (1.08)	0.45	0.65	0.06
Relationship status	1.23	0.54	0.11		
Married	58.0%	58.0%			
Cohabiting	26.9%	26.9%			
Dating	15.1%	15.1%			
Educational attainment	0.89	0.64	0.09		
Higher education	60.3%	60.3%			
Secondary education	32.9%	32.9%			
Primary education	6.8%	6.8%			
Employment status	1.45	0.48	0.12		
Full-time	78.5%	78.5%			
Part-time	12.3%	12.3%			
Not employed	9.2%	9.2%			
Children in household	59.9%	59.9%	0.12	0.73	0.02
EMA compliance rate	84.5% (9.1%)	84.1% (9.3%)	0.32	0.75	0.04

### 3.2 Gender differences in emotional disconnection and emotional loneliness

Descriptive Statistics and Overall Patterns. Across the 3-day EMA assessment period, emotional disconnection scores ranged from 1.0 to 7.0 (M = 3.45, SD = 1.23), and emotional loneliness scores ranged from 1.0 to 7.0 (M = 3.18, SD = 1.34) for the combined sample. Examination of the daily assessment patterns revealed that emotional disconnection and emotional loneliness scores remained relatively stable across the three-day measurement period. On Day 1, emotional disconnection averaged 3.42 (SD = 1.25), and emotional loneliness averaged 3.15 (SD = 1.36). On Day 2, emotional disconnection averaged 3.48 (SD = 1.21) and emotional loneliness averaged 3.21 (SD = 1.33). On Day 3, emotional disconnection averaged 3.45 (SD = 1.24), and emotional loneliness averaged 3.18 (SD = 1.32). These patterns suggest that momentary emotional experiences remained relatively consistent within individuals across the brief assessment window, consistent with previous EMA research on couples’ functioning (Reis et al., 2014).

Gender Differences in Emotional Disconnection and Loneliness. Independent samples t-tests were conducted to examine gender differences in emotional disconnection and loneliness. For emotional disconnection, females reported significantly higher levels (M = 3.62, SD = 1.31) than males (M = 3.28, SD = 1.12; t(198) = 2.41, *p* = 0.017, d = 0.34), which accounted for only 3.4% of the variance in this outcome. For emotional loneliness, females reported significantly higher levels (M = 3.38, SD = 1.42) than males (M = 2.98, SD = 1.24; t(198) = 2.67, *p* = 0.008, d = 0.38), which accounted for only 3.8% of the variance. Following recommendations for reporting effect sizes in mixed-effects models (Nakagawa and Schielzeth, 2012), these modest effect sizes warrant careful interpretation. While these effect sizes are modest and gender accounts for minimal variance, the consistency across both measures suggests a reliable pattern that warrants careful interpretation of the results. These findings indicate that females experience greater momentary emotional disconnection and emotional loneliness within their romantic relationships during the 3-day assessment period than their male counterparts (Maes et al., 2019).

Correlations Between Emotional Disconnection and Emotional Loneliness. Pearson’s correlation analysis was conducted separately for males and females to examine the relationship between emotional disconnection and emotional loneliness within each gender group. For females, emotional disconnection and emotional loneliness were significantly and positively correlated (*r* = 0.68, *p* < 0.001), indicating a strong association between the two constructs. For men, the correlation between emotional disconnection and emotional loneliness was also significant and positive (*r* = 0.62, *p* < 0.001), suggesting a moderate-to-strong relationship between the two. The correlation coefficient for females was slightly higher than that for males, although both correlations were substantial and statistically significant. These findings suggest that individuals who experience greater emotional disconnection from their partners tend to simultaneously experience higher levels of emotional loneliness within the romantic relationship, and this pattern holds true for both genders, with a somewhat stronger association in women (Anyan and Hjemdal, 2020).

Summary of Gender Differences Patterns. Across both measured constructs—emotional disconnection and emotional loneliness—females consistently reported higher levels than males. Gender differences in emotional disconnection accounted for approximately 3.4% of the variance (d = 0.34), and gender differences in emotional loneliness accounted for approximately 3.8% of the variance (d = 0.38). Despite these modest effect sizes, the gender differences were statistically significant and replicated across both outcome measures, suggesting a consistent pattern of greater emotional disconnection and loneliness among female partners in this sample of romantic couples (Weber and Hülür, 2021). The strong positive correlations between emotional disconnection and emotional loneliness within each gender underscore the conceptual and empirical overlap between these constructs in intimate relationships.

## 4 Discussion

This study examined gender differences in emotional disconnection and emotional loneliness among romantic couples using a 3-day ecological momentary assessment (EMA). The findings revealed significant gender differences in both constructs, with females reporting higher levels of emotional disconnection and loneliness than males (Table 2). Specifically, females exhibited higher emotional disconnection (M = 3.62, SD = 1.31) than males (M = 3.28, SD = 1.12), t(198) = 2.41, *p* = 0.017, d = 0.34, and similarly higher emotional loneliness (M = 3.38, SD = 1.42) than males (M = 2.98, SD = 1.24), t(198) = 2.67, *p* = 0.008, d = 0.38. These findings contribute to the growing body of literature documenting gender differences in emotional experiences in intimate relationships (Boyacioglu et al., 2017). The effect sizes, while modest, were statistically significant and consistent across both outcome measures, suggesting a robust pattern of differential emotional experiences between the sexes in romantic relationships.

**Table 2:** Gender differences in emotional disconnection and emotional loneliness and correlations (*N* = 100 romantic couples).

Variable	Females M(SD)	Males M(SD)	t	df	*p*	Cohen’s d	Females-males correlation
Emotional disconnection	3.62 (1.31)	3.28(1.12)	2.41	198	0.017*	0.34	
Emotional loneliness	3.38 (1.42)	2.98 (1.24)	2.67	198	0.008*	0.38	
Females: Emotional disconnection ↔ Emotional loneliness							r = 0.68***
Males: Emotional disconnection ↔ Emotional loneliness							r = 0.62***

*p < 0.05; ***p < 0.001. Effect size (Cohen’s d) was interpreted as follows: small (d ≤ 0.20), small-to-medium (0.20 < d ≤ 0.50), medium (0.50 < d ≤ 0.80), and large (d > 0.80). The Pearson correlation coefficients are presented separately for women and men. All correlations between emotional disconnection and emotional loneliness were significant (*p* < 0.001).

The observed sex differences in emotional disconnection and loneliness warrant careful theoretical consideration and may reflect multiple mechanisms. Research has consistently demonstrated that women tend to report greater emotional expressivity and relational focus than men, particularly in romantic relationships (e.g., Simpson et al., 2007). From an emotion socialization perspective, women’s socialization toward relational maintenance may heighten cognitive and affective attunement to subtle shifts in harmony, creating a threshold effect wherein women perceive and report disconnection more readily than men (Dobson et al., 2022). Alternatively, from an attachment theory perspective, women’s stronger disconnection-loneliness correlation (*r* = 0.68 vs. *r* = 0.62) suggests that anxious attachment mechanisms may be more readily activated in response to perceived partner distance (Schmitt, 2003). Conversely, men’s lower reported levels of emotional disconnection could reflect either genuinely lower subjective experiences of disconnection or a tendency toward emotional suppression and avoidance, which are patterns sometimes associated with masculine socialization (Ryan et al., 2005). The parallel gender differences in emotional loneliness suggest that women’s heightened emotional need for connection and understanding within a relationship may render them more vulnerable to loneliness, even in the presence of a partner.

The strong positive correlation between emotional disconnection and emotional loneliness within each gender (females: *r* = 0.68, *p* < 0.001; males: *r* = 0.62, *p* < 0.001; see Table 2) indicates a substantial overlap between these constructs. The correlation between emotional disconnection and emotional loneliness was slightly stronger in females (*r* = 0.68) than in males (*r* = 0.62). Although statistically significant (z = 1.32, *p* = 0.047), the practical magnitude of this difference is modest (Δr = 0.06). This finding aligns with attachment theory, which proposes that anxious attachment styles—more prevalent among women—are characterized by heightened sensitivity to partner availability and emotional responsiveness (Schmitt, 2003). The strong interdependence between emotional disconnection and emotional loneliness in females may reflect the operation of attachment anxiety mechanisms, in which perceived emotional distance triggers acute emotional loneliness.

The stability of emotional disconnection and emotional loneliness across the 3-day assessment period (Table 2) suggests that these experiences showed consistency over this brief monitoring window, indicating that momentary emotional states reflect underlying relationship characteristics rather than fleeting situational fluctuations. Mean levels remained relatively consistent across Days 1, 2, and 3, suggesting that momentary emotional states within this domain reflect more enduring relationship characteristics than situational fluctuations. The stability observed over the 3-day period does not preclude substantial within-person variability over longer timescales or in response to specific relationship events, which warrants investigation in future research. This finding is consistent with prior EMA research, which demonstrates that relationship-based emotional experiences show considerable stability within individuals over short periods of time (Langer et al., 2018). However, the stability observed over the 3-day period does not preclude substantial within-person variability over longer timescales or in response to specific relationship events, which remains an important avenue for future research.

Several methodological strengths of the present study enhance confidence in the findings and reveal advantages that extend considerably beyond simply documenting between-group differences. The EMA methodology provides ecological validity by capturing emotional experiences in real-world contexts rather than relying on retrospective recall, thereby reducing memory bias and enhancing the accuracy of the reported emotional states (Triantafillou et al., 2019). Critically, the six daily assessments across three consecutive days generated 18 momentary snapshots per individual, revealing within-person patterns of emotional variability that illuminate how disconnection and loneliness fluctuate relative to each person’s baseline, rather than solely comparing group averages. The high compliance rate (84.3%) demonstrates participant engagement and data quality. The dyadic design, with simultaneous assessment of both members of each couple, provides methodological advantages for examining gender differences while accounting for the non-independence of observations within couples through appropriate multilevel modeling (Kenny and Kashy, 2011). The use of distinguishable dyads (heterosexual couples) with gender as the primary organizing variable aligns with standard practices in dyadic data analysis.

Despite these strengths, the present study has some notable limitations that warrant discussion. First, and most critically, the modest effect sizes warrant careful consideration. Gender accounted for only 3.4–3.8% of the variance in these outcomes, leaving over 96% unexplained by gender. This raises important questions about practical significance: if one randomly selected a man and woman from the sample, the probability of correctly predicting who reported higher disconnection based on gender would be marginally better than chance. These small effects may reflect measurement artifacts or gender-differentiated response styles rather than genuine phenomenological differences. Clinically, practitioners cannot reliably use gender to predict individual clients’ emotional experiences, highlighting the necessity for individualized assessment rather than gender-stereotyped assumptions. Second, the 3-day assessment window, while sufficient for capturing momentary emotional experiences and reducing participant burden, limits the ability to examine longer-term patterns and potential cyclical fluctuations in emotional disconnection and loneliness across extended time scales. Third, the sample primarily consisted of cohabiting or married couples in relatively long-term relationships (M = 7.3 years), which may limit the generalizability of the findings to dating couples, newly formed relationships, or diverse relationship structures such as same-sex couples or long-distance relationships. Fourth, the study employed self-report measures, which are subject to social desirability bias and potential gender-specific response patterns. For example, men may systematically underreport their emotional experiences because of cultural norms regarding emotional expression, potentially inflating apparent gender differences. Fifth, the cross-sectional nature of the 3-day assessment precludes causal inferences regarding the antecedents and consequences of emotional disconnection or loneliness.

Future research should extend this line of inquiry in several ways, as follows. Longitudinal EMA studies spanning weeks or months would enable the examination of temporal dynamics, potential cyclical patterns, and stability of gender differences across extended periods. Specifically, future work should investigate whether gender differences reflect genuine differences in experienced disconnection or gender-differentiated awareness and reporting of equivalent underlying disconnection, employ multi-method assessment (behavioral observation, physiological measures, partner reports) to validate self-report differences, and examine how attachment anxiety and avoidance dimensions, emotion regulation strategies, and communication patterns mediate these gender differences. Additionally, such mechanistic investigations could transform our understanding from documenting “that” gender differences exist to explaining “why” and “how” they operate within daily couple interactions. Including diverse relationship types, such as same-sex couples and long-distance relationships, would enhance the generalizability of the findings and potentially reveal how relationship structures moderate gender differences in relationship maintenance behaviors. Intervention studies employing couple-based therapies targeting emotional disconnection and loneliness could test whether gender-differentiated interventions are necessary or whether uniform approaches effectively address these experiences for both partners. Additionally, integrating physiological measures (e.g., heart rate variability and cortisol) with self-reported emotional experiences could provide a more comprehensive understanding of the embodied nature of emotional disconnection and loneliness across sex.

Clinical and Theoretical Implications. These findings carry several important implications for couple therapy and relationship science. Clinically, the stronger disconnection-loneliness link in women suggests that female partners’ heightened reports of emotional distress should not be dismissed as over-sensitivity. However, whether these momentary patterns predict long-term relationship deterioration requires longitudinal investigation. Therapists should consider gender-differentiated intervention strategies: for women showing elevated disconnection and loneliness, interventions targeting enhanced partner empathic attunement and responsiveness may be particularly effective, whereas for male partners, interventions addressing emotional awareness and expression capacity may be more appropriate. Theoretically, these findings underscore that gender does not simply determine relationship experiences uniformly but rather shapes the specific mechanisms and pathways through which relational processes influence emotional states.

In conclusion, this study provides evidence of statistically significant gender differences in momentary emotional disconnection and emotional loneliness within romantic couples, with women consistently reporting higher levels than men. However, given that gender explains only 3–4% of the variance in these experiences, the practical significance of these differences remains to be established. The strong correlation between these constructs and the stability of experiences across the 3-day assessment period underscores their relevance as important dimensions of couple functioning. These findings have implications for relationship counseling and psychotherapy, suggesting that clinicians should recognize gender as one of many factors influencing relationship emotional experiences and emphasize individualized assessment rather than gender-based assumptions when working with couples. Most importantly, future research should employ multi-method measurement, investigate underlying mechanisms, and examine whether gender differences actually predict relationship outcomes or treatment response—questions central to establishing whether observed gender differences matter for real-world clinical and relational consequences.

## Data Availability

The original contributions presented in the study are included in the article/supplementary material, further inquiries can be directed to the corresponding author.
